# Reduction of the nematode egg reappearance period in horses after anthelmintic therapy

**DOI:** 10.14202/vetworld.2022.1530-1534

**Published:** 2022-06-24

**Authors:** Maria V. Baranova, Olga A. Panova, Daria N. Polukhina, Daria S. Panova

**Affiliations:** Laboratory of Biology and Biological basis of Preventive Measures, Federal State Budget Scientific Institution “Federal Scientific Centre VIEV” (FSC VIEV), St. B. Cheryomushkinskaya, 28, Moscow, 117218, Russia

**Keywords:** anthelmintic resistance, eggs reappearance period, horse parasitic nematodes, *Parascaris equorum*, strongyles

## Abstract

**Background and Aim:**

Anthelmintics are used to control equine nematodes. However, helminth resistance to regularly used drugs is a well-known challenge. Among tests to assess effective control and monitor resistance, the most common is the fecal egg count reduction test (FECRT). In the absence of reliable FECRT results, the nematode egg reappearance period (ERP) is taken into account. This study aimed to examine horses from farms around the Moscow Region to assess nematode resistance through ERP after therapy.

**Materials and Methods:**

In the first stage, fecal samples from 280 horses were examined by the flotation method with a sodium nitrate solution. The eggs per gram (EPG) in feces were counted using the modified McMaster technique. One hundred and forty out of 280 horses were selected for further work. Five groups were formed: Two groups of horses infected with strongyles (n = 50) and three groups with *Parascaris equorum* (n = 90). Therapy against strongyles was performed with albendazole and ivermectin. Therapy for parascaridosis was performed with fenbendazole, ivermectin, and aversectin C. Samples from the horses in each group were taken on the 14^th^ day (2 weeks), 28^th^ day (4 weeks), 42^nd^ day (6 weeks), 56^th^ day (8 weeks), and 84^th^ day (12 weeks) after treatment, and the amount of EPG in each sample was determined.

**Results:**

Overall, nematodes were found in 65% of the horses examined. *P. equorum* was most frequently recorded (42.1%) followed by *Strongylidae* gen. spp. (27.9%). The strongyles ERP after therapy with albendazole and ivermectin was estimated on 42 days (6 weeks). The growth of *P. equorum* eggs in the feces was observed from the 56^th^ day (8 weeks) after therapy with fenbendazole, from the 42^nd^ day (6 weeks) after therapy with ivermectin, and was observed from the 84^th^ day (12 weeks) after the use of aversectin.

**Conclusion:**

Our study shows widespread reductions in nematode ERPs across the Moscow Region after ivermectin therapy in horses, suggesting that additional monitoring of these farms is needed for effective control of anthelmintic resistance.

## Introduction

Parasitic diseases of horses are widespread in the Russian Federation. Horses are most commonly infected by nematodes, gastrointestinal strongyles, and *Parascaris equorum*, and less often by cestodes, *Anoplocephala magna* and *Anoplocephala perfoliata*, and protozoa *Eimeria leuckarti*. Combined infestations are often recorded [1–8]. In recent decades, the treatment and control of gastrointestinal nematodes in horse breeding have relied heavily on anthelmintics, including such drugs as phenothiazine, piperazine, pyrantel tartrate, pyrantel pamoate, albendazole, fenbendazole, thiabendazole, oxfendazole, avermectins, and ivermectin. The most widely used of these drugs belong to the following chemical classes: Benzimidazoles, tetrahydropyrimidines, and macrocyclic lactones (MLs). However, prolonged use of the same drugs leads to parasite resistance, resulting in decreased efficacy of such therapeutic measures [9–11].

Information on the resistance of equine nematodes to the action of anthelmintics in Russia is currently insufficient. Various tests have been described to detect anthelmintic resistance (AR) both *in vivo* (the fecal egg count reduction test) and *in vitro* (the egg hatch assay test, larval development assay, larval migration inhibition assay, etc.) [12]. Recently, a reduction in the nematode egg reappearance period (ERP) after anthelmintic therapy has been taken into account as an early premonitory sign of resistance. This measure indicates the earliest sign of a change in the helminth population susceptibility to anthelmintic drugs [13, 14]. Information about the early warning signs of a decrease in the effectiveness of anthelmintic drugs is important for horse farms for the timely rotation of anthelmintic drugs.

Therefore, this study aimed to investigate the nematode ERP after anthelmintic therapy.

## Materials and Methods

### Ethical approval and Informed consent

The study protocol was reviewed and approved by the scientific and methodological commission of VNIIP – a branch of the Federal State Budget Scientific Institution “Federal Scientific Center VIEV” (Protocol No. 2 dated February 12, 2020). The procedures used in this study are in line with the principles of the Declaration of Helsinki and the European Convention for the Protection of vertebrate animals used for experimental and other scientific purposes. The heads of horse farms were informed about the purpose and order of the research. They agreed to participate in the study and gave information about the farm, the conditions of animals, the timing of deworming, and the type of drugs.

### Study period and location

The study was conducted in autumn from August to October 2020 and from August to October 2021. The study included 280 horses from six farms located in the Moscow Region: City of Khimki, Istrinsky, Voskresensky, Odintsovsky, Lyubertsy, and Dmitrovsky Districts.

### Animal selection criteria

The work was carried out on farms in which AR was not reported the previous spring. A total of 280 horses were included in the study, both males and females, ranging in age from 1 to 10 years. All animals were grazed during summer and were kept under similar conditions. The horses had not received anthelmintic drugs for at least 3 months before the study.

### Experimental design

The work was carried out in two stages. In the first stage, fecal samples from 280 horses were studied by the flotation method. Based on the results from this stage, 140 horses found to be infected with parasites were selected for the second stage, 50 infected with strongyles and 90 infected with *P. equorum*. All horses were infected with one type of nematode and did not have a mixed parasitic infestation. Five experimental groups were formed. Groups 1 and 2 included horses with strongyles, with the number of eggs per gram (EPG) in feces ≥ 225. Group 1 (n = 35) received a drug containing albendazole as the active substance at a dosage of 7.5 mg/kg. Group 2 (n = 15) received ivermectin at a dose of 200 mg/kg. Groups 3–5 included horses infected with *P. equorum*, with EPG ≥ 175. Group 3 (n = 20) received fenbendazole at a dose of 7.5 mg/kg. Group 4 (n = 40) received ivermectin at a dose of 200 mg/kg. Group 5 (n = 30) was administered a drug which included aversectin C, at a dosage of 250 mg/kg. All drugs were administered orally, once. Body weights were determined with a girth tape or using platform scales. Fecal samples were taken from all horses before anthelminthic treatment (day 0), on 14, 28, 42, 56, and 84 days after treatment (corresponding to 0, 2, 4, 6, 8, and 12 weeks, respectively).

### Collection and examination of fecal samples

Fecal samples were taken from all animals directly from the rectum. The samples were examined in the laboratory by the flotation method with a sodium nitrate solution (NaNO_3_, specific gravity [SG] 1.38 g/cm^3^) on the sampling day. The study included centrifugation at 120 g for 3 min (laboratory centrifuge Elmi SM-6M, Latvia) [15]. EPG were counted using the modified McMaster technique. To do this, a counting chamber with a capacity of 0.15 mL was used. Sodium nitrate solution (NaNO_3_, SG 1.38 g/cm^3^) was used as a flotation fluid. We mixed 4 g of feces with 26 mL of the flotation fluid to make a total volume of 30 mL, filtered this through a sieve, immediately filled both chambers with the suspension, and left it for 10 min. To determine EPG, the obtained result was multiplied by 25 [15, 16]. The microscopy was performed on a conventional microscope (Motic BA210, Hong Kong) equipped with a digital camera and associated software for photo documentation and object measurement. The study and quantitative calculations were carried out at 100×. All calculations and statistical data analysis were performed using Microsoft Excel and IBM SPSS v.26.0 software (IBM Corp., NY, USA).

## Results

Of the 280 horses examined, 182 (65.0%) were infested with nematodes (Table-1). These infestations included *Strongylidae* gen. spp. (27.9%) and *P. equorum* (42.1%), with 28 samples (10%) in which multiple types of parasitic nematodes were present as an association.

**Table 1:** Distribution of horse nematode in central Russia (n = 280).

Nematode	Number of positive samples	With eggs, %	Mean EPG (min-max)
*Strongylidae* gen. spp.	78	27.9	353.2 (50–825)
*Parascaris equorum*	118	42.1	274.6 (25–700)
In total	182	65.0	–

In the first group, strongyles therapy was performed with albendazole. About 2.9% of samples were positive on days 14 and 28; 5.6% on day 42; 8.6% on day 56; and 14.3% on day 84. An increase in positive samples was noted from the 6^th^ week. The number of EPG in feces increased from the 6^th^ week, from 37.5 epg at week 6 to 45 epg at week 12 (Table-2). The second group of horses with strongyles received ivermectin. We noted that on the 14^th^ day after therapy, 40% of the samples were positive. The count of EPG in feces was 37.5, which was lower than before therapy. However, after 28 days (4 weeks), no strongyles eggs were found at all. On 42 days (6 weeks), strongyles eggs were recorded in 6.6% of samples. On the 56^th^ and 84^th^ days, an increase in contaminated samples was noted, from 20.0% to 66.6% of samples. The EPG in feces increased from 50.0 on day 42 to 63.9 on day 84 (Table-2).

**Table 2:** Dynamics of reappearance of nematode eggs in horses after anthelmintic therapy.

Group No.	Age, years	0 day		14 days a.t. (2 weeks)		28 days a.t. (4 weeks)		42 days a.t. (6 weeks)		56 days a.t. (8 weeks)		84 days a.t. (12 weeks)	
					
		Number of positive samples (percent, %)	EPG counts, average (min-max)	Number of positive samples (percent, %)	EPG counts, average (min-max)	Number of positive samples (percent, %)	EPG counts, average (min-max)	Number of positive samples (percent, %)	EPG counts, average (min-max)	Number of positive samples (percent, %)	EPG counts, average (min-max)	Number of positive samples (percent, %)	EPG counts, average (min-max)
Strongylide therapy													
1	1–10	35 (100)	406.4 (225–850)	1 (2.9)	25	1 (2.9)	25	2 (5.7)	37.5 (25–50)	3 (8.6)	58.3 (50–75)	5 (14.3)	45 (25–75)
2	2–10	15 (100)	485 (250–850)	6 (40)	37.5 (25–75)	0	0	1 (6.6)	50	3 (20)	41.6 (25–50)	9 (66.6)	63.9 (25–100)
*P. equorum* therapy													
3	2–10	20 (100)	276.2 (175–425)	0	0	0	0	0	0	2 (10)	25 (25)	5 (25)	50 (25–75)
4	1–10	40 (100)	321.7 (175–525)	1 (2.5)	25	0	0	2 (5)	37.5 (25–50)	2 (5)	37.5 (25–50)	25 (62.5)	66 (25–150)
5	2–3	30 (100)	313.1 (200–550)	2 (6.6)	37.5 (25–50)	0	0	0	0	0	0	17 (56.6)	69.1 (25–150)

Days a.t.=Days after therapy.

After treatment against *P. equorum* with fenbendazole (Group 3), all horses in the group were free from *P. equorum* on the 14^th^, 28^th^, and 42^nd^ days (weeks 2, 4, and 6). On the 56^th^ day, 10% of the samples were positive and on the 84^th^ day – 25%. The number of EPG of feces increased to 50 epg by the 84^th^ day. Group 4 was treated with ivermectin for equine parascaridosis. On the 42^nd^ and 56^th^ days (6 and 8 weeks), 5% of the samples were positive, and on the 84^th^ day – 62.5%. EPG of feces increased from 37.5 (day 42) to 66 epg (day 84). After application of aversectin C to horses with *P. equorum* (Group 5), eggs were found in feces on the 84^th^ day post-treatment (12 weeks) in 56.6% of samples. The number of EPGs was 69.1 (Table-2).

## Discussion

Gastrointestinal nematodes remain one of the major health-related challenges of horse breeding and can cause significant economic losses. These losses are associated with a decrease in weight gain of young animals due to poor feed payment, loss of productivity and a decrease in the reproductive function of the thoroughbred, increased susceptibility to infectious diseases, and, in some cases, death of animals [2, 4, 5, 14, 17]. Often, practical veterinary specialists have difficulties in choosing an effective and safe anthelmintic drug. As a result, they are guided by the principle of economic benefit of using drugs with one active substance for a long time. The world literature includes a lot of data on AR prevalence in horse breeding. This problem has been identified and investigated since the middle of the 20^th^ century when phenothiazine resistance was discovered in equine cyathostomins [11].

The cyathostomin resistance is most often determined to benzimidazole [18]. A 2014 review article collected reports about AR cyathostomins to benzimidazole in 13 countries: Australia (2002), Brazil (2008), Canada (1977), Denmark (1991, 1998), France (2012, 2013), Germany (2004, 2009), Italy (2007, 2009), Norway (1995), Slovak Republic (2000, 2009), Sweden (1989, 2007), Ukraine (2008), the UK (1992, 2006, 2009, 2013, 2014), and the USA (eight reports from 1981 to 2013) [14]. The 2019 survey study already included reports from Ethiopia (2017), Nigeria (2018), India (2016), Scotland (2014), and Switzerland (2005) [11].

However, AR to pyrantel is considered less common in cyathostomins, having been first recorded in 1996. Pyrantel resistance was initially reported in North America and was thought to be related to the use of pyrantel tartrate in the US and Canada, which was administered daily with the feed at low doses [18, 19]. However, there are currently many reports of pyrantel resistance in Europe as well [14]. Thiabendazole resistance in cyathostomins was recorded a few years after its discovery, as well as in Norway, Slovakia, and Germany [11]. Views are mixed on the distribution of AR in cyathostomins to MLs. A study concluded that there was no convincing evidence for cyathostomin resistance to MLs [20]. However, a decrease in the effectiveness of therapy for ML cyatostominosis was later registered [21, 22]. Cases have been described for decreased efficacy of ivermectin in cyathostomins in Brazil, Finland, Italy, and the UK [11].

The recording of AR in *P. equorum* has begun recently; however, the resistance, for example, to the MLs in *P. equorum* is currently observed more often than in cyathostomins. *P. equorum* resistance to pyrantel has only been described in the US on a limited number of farms and may also be associated with daily low dose pyrantel with the feed. One study reported a clear lack of pyrantel efficacy against *P. equorum* [11, 14]. The fenbendazole resistance in *P. equorum* was described in France, Great Britain, Saudi Arabia, and other countries [11]. *P. equorum* resistance to MLs was first described in the Netherlands in 2002, followed by data on resistance to MLs in parascarids of horses in Canada, Denmark, the USA, Finland, France, Germany, Italy, Sweden, and Great Britain [11, 14, 23, 24]. There are a growing number of reports describing the reduced moxidectin therapy efficacy against *P. equorum*. There is evidence of *P. equorum* resistance to ivermectin in Canada [11, 19, 25].

A reduction in the ERP after treatment of horse nematodes is considered a premonitory sign of AR. After the treatment of equine cyatostominosis with ivermectin, reductions ERP have been reported in Brazil, Germany, the United Kingdom, and the United States. An ERP reduction after moxidectin treatment in Brazil, the UK, and the US also suggests a possible resistance development [13, 26–28]. *P. equorum* ERP reduction after the MLs treatment was detected in a number of countries [23, 29]. Our study used the following benzimidazoles: Albendazole against strongyles and fenbendazole against equine parascaridosis. Albendazole against strongyles showed ERP after the therapy in the 6^th^ week (day 42). Fenbendazole against *P. equorum* showed ERP from the 8^th^ week (day 56). Previously, data were obtained on *Parascaris* eggs that appeared in feces of the horses 8 weeks after treatment with fenbendazole [9]. Thus, there was no ERP reduction on these horse farms.

Lyons *et al*. [27] described that the ERP after the MLs treatment against equine nematodes was 8–10 weeks. It was 8 weeks after ivermectin treatment and about 10 or more weeks after moxidectin. The studies conducted in Germany described ERP reduction for strongyles with the ivermectin therapy and it was 5 weeks in 2007 [13, 27]. In our study, strongyles and *Parascaris* eggs detected in the horses after ivermectin were observed from the 6^th^ week (42^nd^ day). After aversectin C, *P. equorum* eggs appear only from the 12^th^ week (84^th^ day). Thus, we note a reduction ERP after ivermectin against both strongyles and parascaridosis. This requires further monitoring of these farms and AR control. Such a picture of an ERP reduction for nematodes after ivermectin used in horses is quite typical for horse farms where such drugs are used for a long time. Monitoring the effectiveness of anthelmintic agents may be useful in controlling the development of AR in horse farms.

## Conclusion

Our research has shown that 65% of horses are infected with nematodes. *P. equorum* was more frequently recorded (42.1%) and *Strongylidae* gen. spp. were found less frequently (27.9%). The strongyles ERP after therapy with albendazole and ivermectin showed 6 weeks (42 days). *P. equorum* ERP after therapy fenbendazole were 8 weeks (56 days). After therapy with ivermectin, *P. equorum* ERP were 6 weeks (42 days); after the use of aversectin, the most stable result was achieved at 12 weeks (84 days). Our study shows the nematode ERP reduction after ivermectin therapy in horses. These data indicate the need to monitor AR of horse nematodes, and the effectiveness of anthelmintic drugs should be regularly monitored. More research is needed on horses with different AR statuses to determine the factors critical for AR development in Russia.

## Authors’ Contributions

OAP and MVB: Conception and designed the study. MVB, DNP, and DSP: Collected the samples and data analysis. OAP, MVB, and DNP: Drafted the manuscript. All authors have read and approved the final manuscript.
